# Residue level, occurrence characteristics and ecological risk of pesticides in typical farmland-river interlaced area of Baiyang Lake upstream, China

**DOI:** 10.1038/s41598-022-16088-4

**Published:** 2022-07-14

**Authors:** Xiaoli Sun, Miao Liu, Jianwei Meng, Liping Wang, Xiaoxin Chen, Shan Peng, Xin Rong, Lei Wang

**Affiliations:** 1 Hebei Key Laboratory of Close-to-Nature Restoration Technology of Wetlands, School of Eco-Environment, Hebei University, Baoding, 071002 Hebei Province People’s Republic of China; 2 College of Chemistry and Environmental Science, Hebei University, Baoding, 071002 Hebei Province People’s Republic of China; 3 Hebei Key Laboratory of Mineral Resources and Ecological Environment Monitoring, Hebei Research Center for Geoanalysis, Baoding, 071002 Hebei Province People’s Republic of China

**Keywords:** Environmental monitoring, Environmental chemistry, Environmental impact

## Abstract

Baiyang Lake is the largest freshwater lake in North China, playing an important role in aquatic products production and eco-environment improvement. Traditional organochlorine pesticides were not enough to reflect ecological risk. We performed the high-throughput and non-targeted screening to identify the high-residue and wide-distribution pesticides at farmland-river interlaced area. We firstly reported the residue level and spatio-temporal distribution of typical pesticides in soils and waters (SP1–SP13) near Fuhe river in 2020–2021. The mean recoveries of eight pesticides ranged from 79.4 to 129%. The residues were 0.250–3530 ng/L (water) and 2.79 × 10^−3^–647 μg/kg dw (soil), respectively. Thiamethoxam was dominant with the high-residue proportion (HRP) of 53–95% (water, HRP > 50%) and 63–97% (soil, HRP > 60%), respectively. Most of pesticides almost have no significant season-change. The risk quotient (RQ) model results showed that although most pesticides have no aquatic risk (RQ < 0.01), carbendazim and propionazole deserved attention. The individual thiamethoxam at nearly half of the sites exhibited high terrestrial risk (RQ, 1.070–1.682), while propiconazole was at medium risk (SP1, SP2, SP8, and SP9) and high risk (SP12). The RQ_all_ were in the range of 0.4541–3.327 (earthworm), 0.0239–0.4552 (algae), 0.1094–1.103 (aquatic invertabrates), and 0.1657–1.923 (fish), respectively, so co-residue caused joint toxic effect to aquatic invertebrates.

## Introduction

Pesticide is a “double-edged sword”, and their negative effects on human health and ecological environment should not be ignored while applied in agricultural field^1^. The massive research indicated that pesticides have caused varying degrees of pollution to soils, sediment, surface water, groundwater, and even potable water on a global scale^2^. For example, the average residue of tebuconazole in European river reaches the level of 175–200 μg/L^3^, as well as propiconazole in Tengger River Basin in Malaysia is approximately up to 4493 ng/L^4^. Moreover, these environmental exogenous substances (pesticides etc.) will be transformed to various byproducts due to the biological and abiotic actions in environment. Once the pesticides and their toxicological transformation products (TPs) enter the water environment, their will continue to accumulate in the aquatic animals and plants, directly causing subacute toxicity, acute toxicity, chronic toxicity, and other toxic reactions of sensitive aquatic organisms^5,6^. Furthermore, some contaminants with long persistence, diverse action modes and high bioaccumulation factors can accumulate in the human body through the food chain amplification, finally damage the nervous system, and even cause deformity and cancerization^7^.

With the frequent and unreasonable pesticide use, the banned pesticides such as organochlorine and the widely used non-banned pesticides such as organophosphorus and pyrethroid have threatened the ecosystem and human health^8,9^. At present, the research on pesticide residues in environment mainly focuses on persistent organochlorine pesticides (OCPs) originated from historical legacy^10–12^, while the occurrence characteristics, source apportionment, and ecological risk of novel pesticides widely used in agricultural production today are rarely reported. Due to development and promotion of these new pesticides, carbamates, pyrethroids, neonicotinoids etc. will constantly imported and accumulated in soil and water environment with their frequent irrational utilization. For example, nearly 80 chemical pollutants have been detected in 27 sampling points from Beijing and Tianjin, and the residue level of several pesticides (metalaxyl, carbendazol, diuron, and diflubenzuron etc.) are higher than the maximum residue limit of EU drinking water (0.1 mg/L)^13^. In the waters and sediments of Great Lakes tributaries, neonicotinoid insecticides (NNIs) are frequently detected, and the highest detection rate (53%) was pointed to imidacloprid^14^. Therefore, in addition to focusing on the banned persistent pesticides, the residue and ecological risk of new pesticides should also be attached great importance.

Baiyang Lake is the largest freshwater lake in North China, playing an important role in aquatic products production and ecological environment improvement. However, the pesticides directly or indirectly contaminate the large-area farmland soils around the upper reaches of Baiyang Lake, and these pesticides residues in soil further enter the downstream river and lake water through surface runoff, leaching, rain erosion and other ways. Because of overuse and accumulation effect, the agricultural chemicals have a potential impact on water quality and ecological balance of Baiyang Lake. Therefore, it is of great significance to the quality monitoring of water and soil. At present, although there reported the pesticide residue in sediments and aquatic environment of Baiyang Lake and its surrounding rivers, these studies were about OCPs^15,16^. Along with the widely application of new pesticides, the residues of traditional OCPs have not been enough to reflect the ecological risk of Baiyang Lake. Therefore, the non-targeted screening of pesticides and the identification of frequent-detection pesticides in soil and water of typical farmland-river interlaced area will be of great significance in ecological risk assessment.

Aiming at the typical non-point source pollution area in upper reaches of Baiyang Lake, this study will perform the following works: (1) the high-throughput and non-targeted identification of pesticides using high-resolution LC–MS/MS technology combined with the primary and secondary mass spectrometry databases of more than 1300 common pesticides, in order to screen the high-residue and wide-distribution pesticides in soil and water environment on basis of previous pesticide application survey; (2) the quantitative analysis of targeted pesticides in the typical surface water and soil samples at the four seasons in 2020–2021, as well as the clarification of residue level, occurrence characteristics; (3) the risk assessments of aquatic and terrestrial toxicity, as well as genotoxicity based on environmental monitoring data and toxicology data. This study will be conducive to estimate the pollution contribution of pesticide residues to the upper water of Baiyang Lake, it not only provides decision-making basis for environmental management, and further promotes the standardization and institutionalization of pesticide regulation in China, but also provides scientific guidance for non-point source pollution control and ecological restoration in the surrounding area of Baiyang Lake.

## Results and discussion

### Contamination level and characteristic distribution

**Figure 1:**
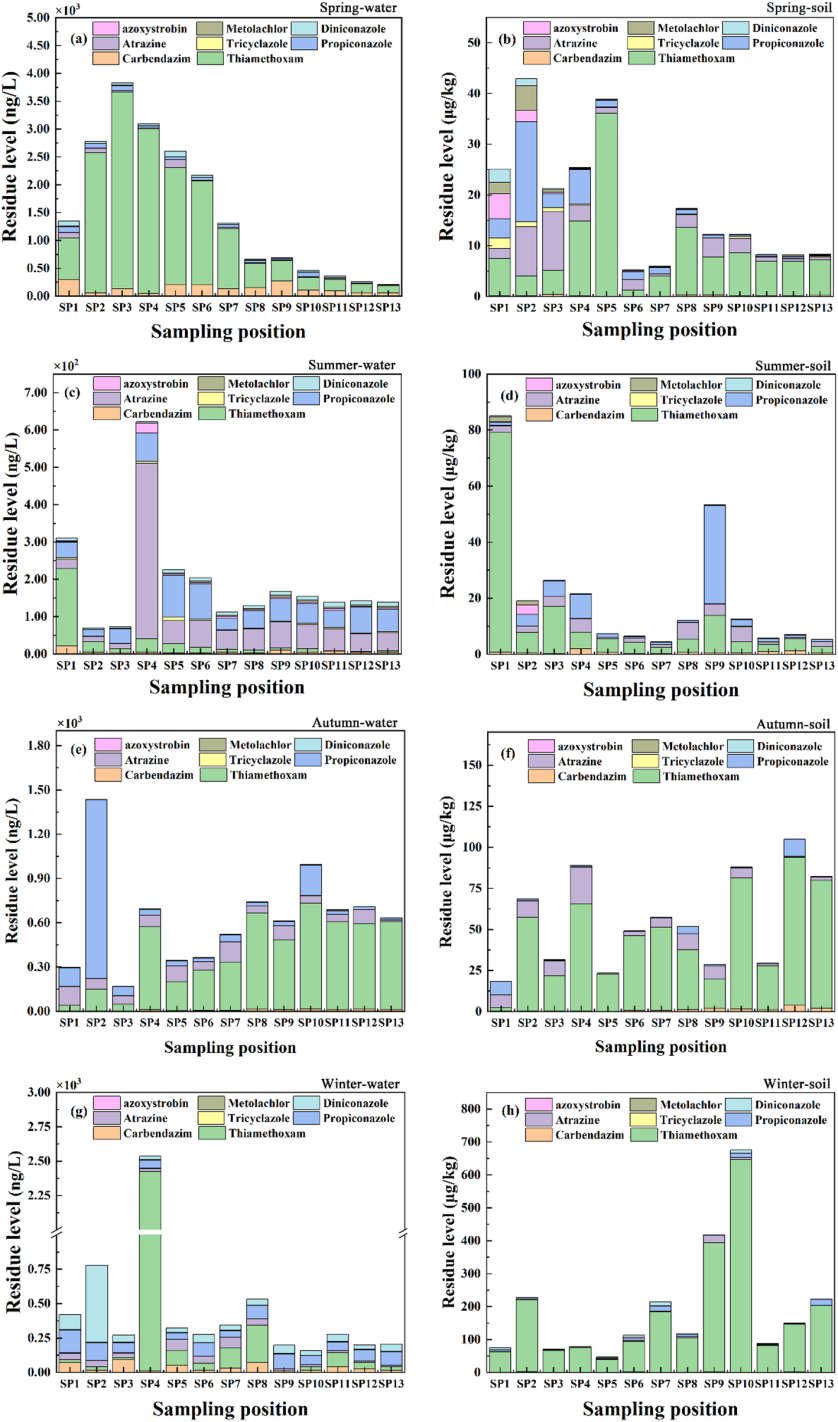
Residue level and range of eight pesticides in 13 sampling positions in different seasons. The residues were 0.250–3530 ng/L (water) and 2.79 × 10^−3^–647 μg/kg dw (soil), respectively. Thiamethoxam was dominant with the high-residue proportion (HRP, thiamethoxam residue/total residue) of 53–95% (water, HRP > 50%) and 63–97% (soil, HRP > 60%), respectively.

During the spring, summer, autumn, and winter in 2020–2021, eight pesticides in all soil and water samples collected from 13 sampling positions were detected with the detection rate of 100%. The residual levels and ranges of these pesticides were showed in Fig. 1a–h, Tables 1 and 2.

**Table 1:** The residue level of eight pesticides in surface waters at 13 sampling positions in the four seasons.

Seasons		Carbendazim	Thiamethoxam	Atrazine	Tricyclazole	Propiconazole	Azoxystrobin	Metolachlor	Diniconazole
Spring	Residue median	132	745	15.8	1.54	46.8	1.56	2.79	32.1
	Mean ± SD	140 ± 80.3	1256 ± 1148	34.9 ± 38.4	1.57 ± 0.806	46.6 ± 30.1	1.74 ± 1.15	4.27 ± 4.19	36.6 ± 26.4
	Range	45.1–302	131–3530	7.46–135	0.754–3.54	3.84–97.4	0.738–5.24	1.00–17.5	5.33–102
Summer	Residue median	4.49	8.60	55.9	2.90	53.9	3.60	3.29	8.82
	Mean ± SD	6.07 ± 4.76	27.7 ± 53.0	80.7 ± 114	3.26 ± 2.15	57.6 ± 24.7	4.94 ± 6.37	3.19 ± 1.50	8.11 ± 2.93
	Range	2.34–21.0	0.715–208	12.5–471	0.489–9.59	17.5–113	0.472–26.4	0.387–5.30	2.85–12.1
Autumn	Residue median	10.1	475	72.3	0.830	33.5	0.612	1.73	0.63
	Mean ± SD	8.55 ± 4.92	400 ± 229	75.1 ± 33.7	1.02 ± 0.465	143 ± 312	0.708 ± 0.314	1.97 ± 1.11	1.18 ± 1.19
	Range	0.867–15.2	41.3–716	13.4–138	0.603–2.29	12.5–1209	0.370–1.53	0.957–5.41	0.250–4.39
Winter	Residue median	23.9	47.6	31.6	1.61	81.3	1.47	2.06	52.8
	Mean ± SD	35.9 ± 26.6	251 ± 628	34.8 ± 23.5	1.64 ± 0.493	86.5 ± 33.1	1.78 ± 1.17	2.02 ± 0.639	88.9 ± 138
	Range	9.23–94.1	2.33–2413	7.38–78.3	0.598–2.80	45.1–164	0.715–5.48	0.963–3.47	23.9–562

They were in the ranges of 0.867–302 ng/L (carbendazim), 0.715–3530 ng/L (thiamethoxam), 7.38–471 ng/L (atrazine), 0.489–9.59 ng/L (tricyclazole), 3.84–1209 ng/L (propiconazole), 0.370–26.4 ng/L (azoxystrobin), 0.387–17.5 ng/L (metolachlor), 0.250–562 ng/L (diniconazole), respectively.

**Table 2:** The average residue level of thiamethoxam in soils.

Seasons		Carbendazim	Thiamethoxam	Atrazine	Tricyclazole	Propiconazole	Azoxystrobin	Metolachlor	Diniconazole
Spring	Residue median	9.20 × 10^−2^	6.97	2.08	0.133	1.20	9.86 × 10^−2^	6.49 × 10^−2^	9.80 × 10^−2^
	Mean ± SD	0.152 ± 0.107	9.37 ± 8.47	3.15 ± 3.38	0.388 ± 0.560	3.05 ± 5.15	0.651 ± 1.37	0.645 ± 1.34	0.396 ± 0.728
	Range	3.25 × 10^−2^–0.398	1.20–36.1	0.467–11.6	5.02 × 10^−2^–2.09	0.199–19.7	4.22 × 10^−2^–4.98	3.17 × 10^−2^–4.88	3.87 × 10^−2^–2.61
Summer	Residue median	0.569	4.74	2.19	6.43 × 10^−2^	1.11	8.59 × 10^−2^	5.21 × 10^−2^	7.49 × 10^−2^
	Mean ± SD	0.663 ± 0.487	11.6 ± 19.8	2.63 ± 1.85	9.48 × 10^−2^ ± 9.86 × 10^−2^	4.76 ± 9.05	0.342 ± 0.874	0.280 ± 0.562	0.101 ± 7.93 × 10^−2^
	Range	0.162–1.99	2.26–78.6	0.527–6.04	1.25 × 10^−2^–0.342	0.451–35.1	4.15 × 10^−2^–3.37	2.26 × 10^−2^–1.73	3.89 × 10^−2^–0.350
Autumn	Residue median	0.640	45.5	5.91	5.27 × 10^−2^	6.17 × 10^−1^	1.73 × 10^−2^	1.81 × 10^−2^	3.29 × 10^−2^
	Mean ± SD	1.05 ± 1.03	45.7 ± 26.1	6.46 ± 5.69	7.10 × 10^−2^ ± 4.32 × 10^−2^	2.20 ± 3.22	1.91 × 10^−2^ ± 9.07 × 10^−3^	3.10 × 10^−2^ ± 3.15 × 10^−2^	4.82 × 10^−2^ ± 3.91 × 10^−2^
	Range	8.21 × 10^−2^–3.91	2.48–89.8	0.432–22.3	3.96 × 10^−2^–0.209	0.121–10.4	8.38 × 10^−3^–3.94 × 10^−2^	7.50 × 10^−3^–0.116	2.17 × 10^−2^–0.175
Winter	Residue median	0.599	104	2.05	1.45 × 10^−2^	3.57	4.14 × 10^−2^	2.21 × 10^−2^	1.78
	Mean ± SD	0.715 ± 0.483	178 ± 163	3.47 ± 5.43	1.72 × 10^−2^ ± 9.79 × 10^−3^	5.77 ± 5.44	0.527 ± 1.48	0.104 ± 0.212	3.34 ± 3.81
	Range	8.97 × 10^−2^–1.79	39.1–647	0.419–21.8	2.79 × 10^−3^–3.63 × 10^−2^	0.404–17.3	3.30 × 10^−3^–5.61	1.06 × 10^−2^–0.825	0.075–12.2

They were in the range of 1.20–36.1 μg/kg dw in spring and 39.1–647 μg/kg dw in winter, whilst other seven pesticides almost no significant fluctuation with the mean residue of below 6.46 μg/kg dw.

#### Surface water

As seen from Fig. 1a, c, e, g, the residual levels of these pesticides in the surface water of four seasons were in the ranges of carbendazim (0.867–302 ng/L), thiamethoxam (0.715–3530 ng/L), atrazine (7.38–471 ng/L), tricyclazole (0.489–9.59 ng/L), propiconazole (3.84–1209 ng/L), azoxystrobin (0.370–26.4 ng/L), metolachlor (0.387–17.5 ng/L), diniconazole (0.250–562 ng/L), respectively. The spatial distribution of eight pesticides in surface water showed that carbendazim and thiamethoxam at spring sampling positions (SP1–SP13) have higher residual concentrations, with the median values of 132 ng/L and 745 ng/L, respectively. Moreover, there appeared their residue peaks as the results of 302 ng/L (carbendazim, SP1) and 3530 ng/L (thiamethoxam, SP3) (Table 1). In summer, atrazine showed the highest residue and detection frequency, appearing its peak concentration (471 ng/L) at SP4 (median value, 55.9 ng/L), followed by propiconazole with the median value of 53.9 ng/L (Fig. 1c). Although atrazine in waters in four seasons was at the trace level with the mean residues of 34.9 ng/L (spring), 80.7 ng/L (summer), 75.1 ng/L (autumn), and 34.8 ng/L (winter), atrazine was listed as one of three carcinogens by WHO (2017), and banned in many countries. In addition, it was reported by Wang et al. that the residue of atrazine in tap water of Northeast China reached up to 122 ng/L^17^. Therefore, atrazine should be highly concerned. As shown in Fig. 1e, the residue of thiamethoxam in the most autumn water samples (SP4–SP13) was dominant with the median concentration of 475 ng/L, whilst propiconazole at SP2 point reached maximum residue (1209 ng/L). In winter, the concentration of diniconazole at SP2 (562 ng/L) was obviously higher than that of other sites (Fig. 1g), while the residue of thiamethoxam at SP4 was far beyond other sites with the highest residue of (2413 ng/L). These results may be related to the seasonal pesticide application or the pesticide discharge to environment, even two common functions^18^.

From seasonal distribution perspective, the residues of thiamethoxam in surface water in spring (mean, 1256 ng/L; median, 745 ng/L) and autumn (mean, 400 ng/L; median, 475 ng/L) were obviously higher than summer (mean, 27.7 ng/L; median, 8.6 ng/L) and winter (mean, 251 ng/L; median, 47.6 ng/L). This result was mainly attributed to pesticide application in seedtime. Compared with other countries, the water pollution induced by thiamethoxam in China was far more serious. For example, the residual mean (median) of thiamethoxam in seven lakes in Vietnam was only 0.81 ng/L (0.43 ng/L)^19^. The average residue of carbendazim in spring was significantly exceeded than that of other season, as the results of spring (140 ng/L), summer (6.07 ng/L), autumn (8.55 ng/L), Winter (35.9 ng/L). Compared with other seasons, diniconazole has higher residue in winter and spring, with the maximum of 562 ng/L and 102 ng/L, respectively. The residues of most pesticides in different seasons almost have no significant change. Among them, tricyclazole, azoxystrobin, and metolachlor were the lower residual, and three pesticides was in the range of 0.49–9.59 ng/L, 0.37–26.42 ng/L, and 0.39–17.48 ng/L, respectively. Besides, the spatio-temporal distribution of eight pesticides were investigated. For most of surface waters from 13 sites in four seasons, the residue of thiamethoxam was dominant than other pesticides, and its high-residue proportion (HRP, thiamethoxam residue/total residue for each sample > 50%) ranged from 53 to 95%. This result may be related to the types of pesticides applied locally. Still further, neonicotinoids are easily transferred into surface water through surface runoff or rain erosion because of its low volatility and water solubility^20^.

#### Soil

Figure 1b, d, f, h and Table 2 showed that the higher-residue pesticides for all soil samples in four seasons were thiamethoxam, atrazine, and propiconazole in proper order, in which the former was more prominent with the high-residue proportion (HRP > 60%) of 63–97%. Thiamethoxam in soil samples exhibited the maximum residue (647 μg/kg dw) at the SP10 site in winter. We found that SP10 site was fruit forest soil, and its higher residue may be caused by application. Meanwhile, it can be found that propiconazole in the spring SP2-soil has higher residue (19.7 μg/kg dw), while the maximum value (35.1 μg/kg dw) appeared in the summer SP9-soil. This was mainly related to agricultural wheat soils (SP2, SP3, SP4, and SP9), because propiconazole has been used to control wheat diseases and insect pests. According to the statistics of eight pesticides in all soil, the lower-residue pesticides were tricyclazole and metolachlor, and the maximum detectable concentration of them were only 2.09 and 4.88 μg/kg dw, respectively. As temporal distribution indicated, the total residue of eight pesticides in most sampling sites has an upward trend from spring to winter. This seasonal dependence-residue was attributing to the temperature-related migration and transformation of pesticides in environment. Usually, the lower temperature in winter leads to slower migration and transformation, so appeared the higher residue in winter. By and large, there was an increase in average residue of thiamethoxam in soils from spring (1.20–36.1 μg/kg dw) to winter (39.1–647 μg/kg dw), whilst almost no significant fluctuation was observed for other seven pesticides, and the mean residue of them was below 6.46 μg/kg dw.

### Toxicity risk of eight pesticides

#### Aquatic toxicity

**Figure 2:**
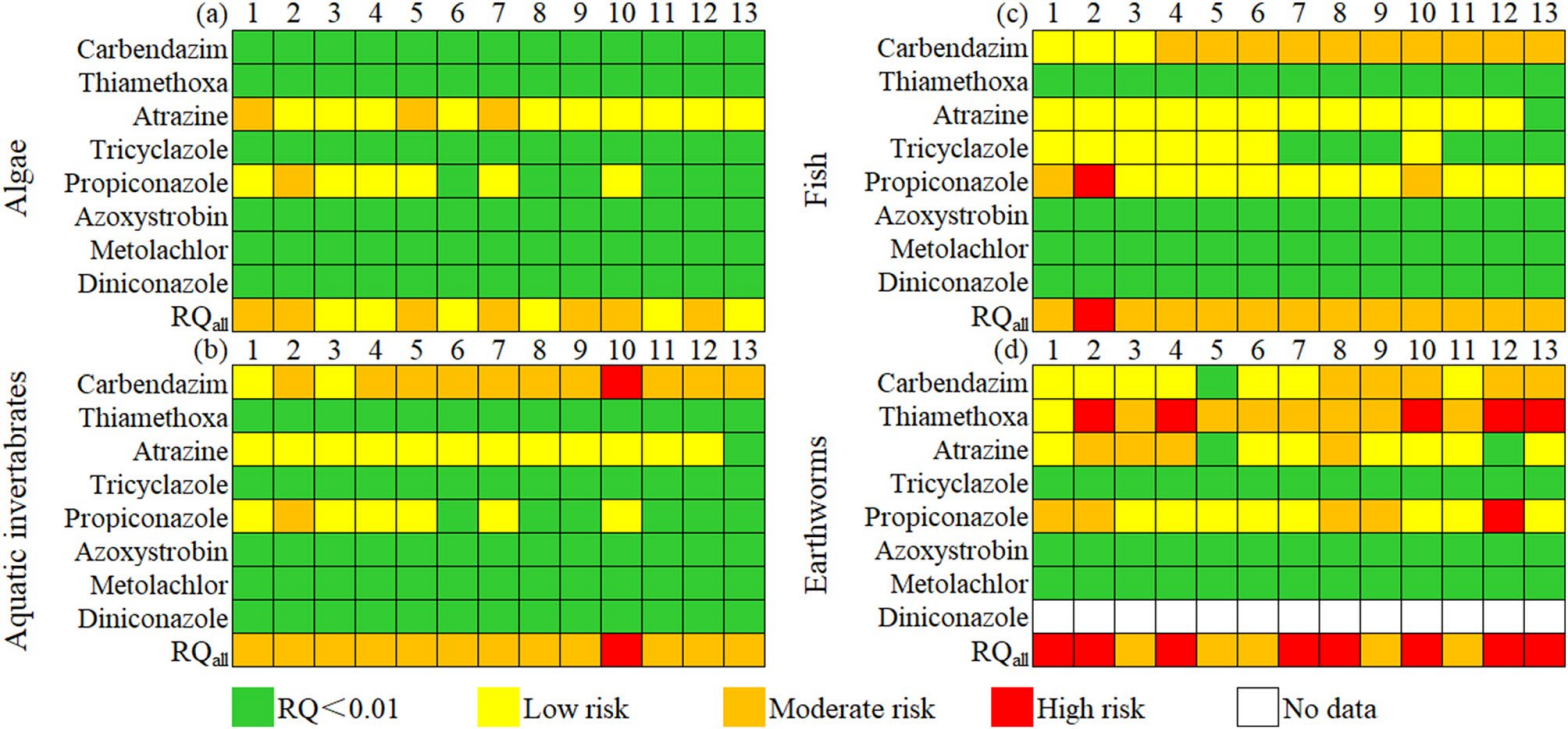
Heat map of RQ values of pesticides to algae (**a**), aquatic invertebrate (**b**), fish (**c**), and earthworm (**d**). Most of pesticides almost have no aquatic risk (RQ < 0.01), but carbendazim and propionazole deserved attention. The RQ_all_ were in the range of 0.4541–3.327 (earthworm), 0.0239–0.4552 (algae), 0.1094–1.103 (aquatic invertabrates), and 0.1657–1.923 (fish), respectively.

The estimated RQ values of eight pesticides in autumn-surface water to algae, aquatic invertebrates, and fish were summarized in Fig. 2a–c, Supplementary Tables 1–3. As shown in Supplementary Table 1, the RQs of carbendazim, tricyclazole, azoxystrobin, metolachlor, and diniconazole to algae were far below 0.01, showing no toxicity risk. The RQs of atrazine in the three waters of SP1 (0.1243), SP5 (0.1068), and SP7 (0.1380) indicated a moderate risk to algae, while low risk at the other sites. Although the maximum RQ of propiconazole was 0.3779 (SP2), it is still a medium risk to algae. Moreover, propiconazole at other waters was low risk even no risk. Compared with other seven pesticides, the carbendazim to aquatic invertebrates should not be ignored, because it was at lower risk only at SP1 and SP3 points, while showed a high risk at SP10 (RQ = 1.013) and moderate risk for most sites (Supplementary Table 2). Besides, we found that the risk of propiconazole in SP2-water was at the moderate level (RQ = 0.3901), while it showed low risk at other sites. Moreover, carbendazim posed a moderate risk (RQ = 0.2071) at the same site. It is worth noting that the risk of propiconazole to algae at SP2 site was at the moderate level. All other pesticides to aquatic invertebrates were low risk. It can be seen from Supplementary Table 3, carbendazim was low risk to fish at SP1 (RQ = 0.0271), SP2 (RQ = 0.0971), and SP3 (RQ = 0.0302), while has moderate risk at the other sites with the RQ range of 0.1294–0.4746. The propiconazole embodied high risk to fish at SP2 (RQ = 1.779), moderate risk at SP1 (RQ = 0.1857) and SP10 (RQ = 0.3044), and no risk at other sites. Therefore, aquatic risk for the above two pesticides deserved attention. The risk of atrazine to fish at the sites of SP1–SP12 was at the low level (0.01 < RQ < 0.1), excepting for SP13 (RQ = 0.0067). In all the 13 surface waters, other five pesticides (tricyclazole, thiamethoxam, azoxystrobin, metolachlor, and diniconazole) showed a low risk or even no risk to fish. The RQ_all_ of eight pesticides to algae, aquatic invertebrates, and fish was displayed in Supplementary Table 4. The RQ_all_ was in the range of 0.0239–0.4552 (algae), 0.1094–1.103 (aquatic invertabrates), and 0.1657–1.923 (fish), respectively. Furthermore, the co-residue of eight pesticides posed a high risk to aquatic invertebrates at site SP10, as well as to fish at site SP2, and both of two cases were mainly contributed by carbendazim and propionazole. These results reflected that the coexistence of multiple pesticides in environment will cause higher ecological risk.

#### Terrestrial toxicity

Selecting earthworms as target organisms, the RQ values of eight individual pesticides and their RQ_all_ were estimated as shown in Fig. 2d and Supplementary Table 5. Evidently, the ecological risk of tricyclazole, azoxystrobin, and metolachlor to earthworms can be ignored because of RQ < 0.01 (or ≪ 0.01). However, there were five and four sites at medium risk for carbendazim and atrazine, respectively, with the other sites at the low level. Among the eight pesticides, individual thiamethoxam exhibited the highest terrestrial risk with only one low-risk site (RQ = 0.0465), and nearly half of the sampling sites (SP2, SP4, SP10, SP12, ad SP13) were at high risk with the RQ value of 1.070–1.682. This result may be due to the higher detection concentration of thiamethoxam. Propiconazole was at medium risk at the four sites of SP1, SP2, SP8, and SP9, and high risk at SP12. RQ_all_ values of eight pesticides ranged from 0.4541 to 3.327, implying that the joint toxic effects should not be ignored.

#### Potential genotoxicity

The potential genotoxicity of surface water was investigated on basis of SOS/umu test in this study. The growth factor (G), induction rate (IR), and potential carcinogenic risk coefficient (P) of 20 times-concentrated surface waters were showed in Supplementary Table 6. Obviously, the IR values for all water samples can be calculated because of G > 0.5. In addition, all the IR values were less than 1.5, which indicated that the bacterial growth was not significantly inhibited. Therefore, these enriched water samples did not mutagenicity against TA 1535/PSK 1002 in the short term to a certain extent. However, the potential carcinogenic risk coefficients of 20 times-enrichment waters varied from 4.82 × 10^−6^ to 5.88 × 10^−6^, which was slightly higher than the referenced safe drinking standard (10^−6^) in this study. In view of bioaccumulation and amplification, these samples may produce carcinogenic risk in the long-term, so there should be paid more attention. Similarly, the G value for SP1 soil sample was below 0.5, probably because the soil extract to bacterial cells has too toxicity (Supplementary Table 7). The extract of SP5 soil was mutagenic positive and genotoxic because the G value (3.98) was beyond 2. At the two sites of SP6 and SP7, the G values were lower than 1.5, indicating no apparent inhibition of bacterial growth. In addition to the above, other G values were in the range of 1.5–2.0, implying that these soil extracts were suspected positive in the mutagenicity.

## Conclusion

Overall, the residues of eight pesticides ranged from 0.250 to 3530 ng/L in surface water and 2.79 × 10^−3^–647 μg/kg dw in soil, respectively. Temporal and spatial distribution results showed that carbendazim, thiamethoxam, and propiconazole were mainly contributed to soil and water pollution. Although atrazine in surface waters for four seasons was at the trace level, the banned pesticide deserves high attention due to frequent detection in many countries. The total residue of eight pesticides has a seasonal dependence that mainly attributed to the temperature-related migration and transformation of pesticides in environment. Carbendazim showed the strongest toxicity to three typical aquatic organisms compared with other seven pesticides, and most pesticides reached a moderate risk level, even a few pesticides have reached high risk level. Propiconazole and carbendazim should be paid more attention to the aquatic ecological risk. What’s more, although the aquatic and terrestrial toxicity of single pesticide was at a low risk level, the RQ_all_ values of eight pesticides in almost all the samples reached medium risk or even more. Therefore, the cumulative effect and joint toxicity of multiple pesticides in environment should be highly concerned in the future.

## Methods

### Nontargeted screening and pre-treatment

Based on the actual investigation of pesticides application and the national monitoring sections, a batch of soil and water samples were collected from the farmland-river cross area in the upper reaches of Baiyang Lake and its entrance through the geographic information system (GIS). The water sample (1.5 L) was enriched and concentrated using MOF-based MSPE method^21^. The traditional QuChERS method was used to pre-treated soil samples (10 g). In order to determine the sampling points with high frequency detection and the kinds of pesticides in these soils and waters, the high-throughput and non-targeted screening of pesticides were performed in the full-Scan and SRM mode by high-resolution LC–MS/MS and GC–MS/MS technology combined with the elementary accurate quality database and the secondary fragment ion spectrogram database.

### Targeted sample collection

**Figure 3:**
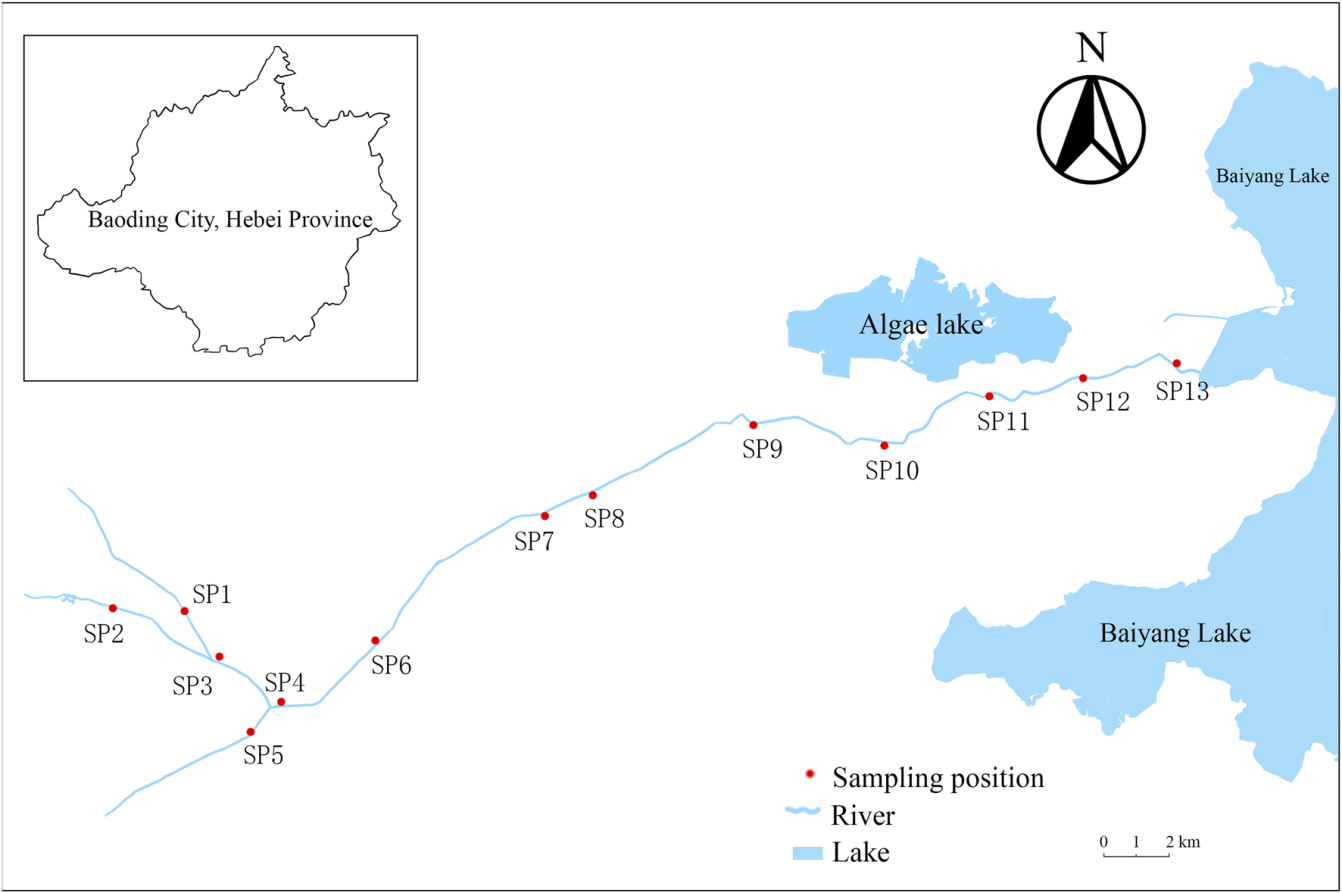
Geographic location map of sampling positions.

Based the result of nontargeted screening, there set 13 sampling points from the upper reaches of Fuhe River to the entrance of Baiyang Lake (Fig. 3 and Supplementary Table 8). In October 2020, December 2020, April 2021, and June 2021, the water samples (below 50 cm from the water surface) were collected using a hydrophore, and the parameters pH, DO, and temperature were measured on site (Supplementary Table 9). The topsoil samples (0–20 cm) were collected from by five-spot-sampling method. These blended samples were brought back to the laboratory as soon as possible. The water samples were refrigerated at 4 °C. The soils were vacuum dried, subsequently ground and sieved through 40 meshes. The pH values of soils were determined by according to the national environmental protection standard of the people's Republic of China (HJ 962-2018). To enrich the target pesticides in water samples, the liquid–liquid extraction (LLE) method was used in this study.

### Method validation

The accuracy and precision of the method were verified in this study. As showed in Table 3, the recoveries of eight pesticides were in the range of 79.4–129%, with the relative standard deviation (RSD) of 4.52–18.8%. The limits of detections (LODs) of eight pesticides in both soil and water were below 1.04 × 10^−2^ μg/L (water) and 0.49 μg/L (soil), respectively, whilst the limits of quantification (LOQs) were below 5.85 × 10^−3^ μg/L for water and 1.63 μg/kg for soil (Supplementary Table 10). The matrix-matched standard curves of eight pesticides in both soil and waters have good linearity (R^2^, 0.9910–0.9992). The above results indicated that this method can be used for monitoring of target pesticides in environmental samples.

**Table 3:** Mean recoveries and relative standard deviation of eight pesticides in soil and waters.

Pesticides	Mean recoveries ± RSD (%, n = 5)					
	Water			Soil		
	0.001 mg/L	0.01 mg/L	0.1 mg/L	0.002 mg/L	0.01 mg/L	0.1 mg/L
Carbendazim	116 ± 6.75	129 ± 5.01	94.9 ± 7.73	96.4 ± 11.0	103 ± 4.93	79.4 ± 10.6
Thiamethoxam	116 ± 9.25	102 ± 12.2	122 ± 6.03	99.0 ± 12.1	102 ± 12.8	98.1 ± 6.35
Atrazine	111 ± 11.5	112 ± 18.3	109 ± 10.4	101 ± 10.5	96.9 ± 11.7	101 ± 7.31
Tricyclazole	118 ± 11.4	118 ± 14.3	117 ± 13.3	123 ± 11.1	79.9 ± 5.18	100 ± 7.79
Propiconazole	101 ± 18.2	111 ± 8.22	111 ± 7.04	95.1 ± 17.2	87.3 ± 4.52	107 ± 9.35
Azoxystrobin	104 ± 6.60	107 ± 18.8	120 ± 8.35	112 ± 9.47	112 ± 10.8	100 ± 9.33
Metolachlor	112 ± 10.2	115 ± 15.6	117 ± 10.1	103 ± 10.5	105 ± 5.41	90.8 ± 8.41
Diniconazole	86.2 ± 5.90	100 ± 7.86	104 ± 6.01	122 ± 5.90	100 ± 11.9	99.9 ± 5.54

Mean recoveries of eight pesticides in soil and waters were in the range of 79.4–129%, and the relative standard deviation (RSD) varied from 4.52 to 18.8%.

### Sample pre-treatment

100 mL water sample was transformed to a triangular bottle with stopper, and added 25 mL CH_2_Cl_2_. Followed by 15 min-ultrasound and 1 min-vortex oscillation, the mixed solution was centrifuged at 4000 rpm for 5 min. The underlayer CH_2_Cl_2_ was transformed into a 100 mL-flask. Another 25 mL CH_2_Cl_2_ was added into the remaining mixture, and repeated the above procedure. The second underlayer CH_2_Cl_2_ was incorporated into the same flask. Subsequently, the vacuum rotary evaporation was carried out by an evaporator under 40 °C water bath. The residues were reconstituted with 0.5 mL acetic acid-acetonitrile mixture (1%, v:v) and analyzed by LC–MS/MS. Analogously, the soil samples (dry weight 10 g) in a 50 mL-PTFE centrifuge tube was extracted 4 ml deionized water and 10 mL 1% acetic acid-acetonitrile (v:v). After vortex extraction for 2 min, 1 g NaCl was added for salting out by vortex oscillates of 30 s, following by the addition of 2 g MgSO_4_ and vortex oscillates of 30 s. The samples were centrifuged at 4000 rpm for 5 min to obtain the supernatant. Afterwards, the 6 mL supernatant was purified by 50 mg PSA. Subsequently, the vacuum rotary evaporation was carried out by an evaporator under 40 °C water bath. The residues were reconstituted with 0.5 mL acetic acid-acetonitrile mixture (1%, v:v) and analyzed by LC–MS/MS.

### Instrument parameters

These pesticides were analyzed in MRM mode of HPLC-QqQ-MS/MS analyser with the ESI +. The mobile phase was the mixture of acetonitrile (A) and 0.2% formic acid aqueous solution (B) with the flow rate of 0.4 mL/min. The mass parameters of pesticides and elution procedure were showed in Supplementary Tables 11–12. The injection volume was 10 µL using methanol as washing liquid. The column temperature and ion source temperature were controlled at 30 °C and 200 °C, respectively. N_2_ was used as nebulizer with 40 psi, 450 °C, and 11 L/min. The capillary voltage was controlled at 4000 V.

### Recovery trials

The appropriate amount of standard mixture (50 mg/L or 5 mg/L) was added into 100 mL blank actual surface water uncontaminated by the tested eight pesticides to reach the spiked level of 0.1 mg/L, 0.01 mg/L, and 0.001 mg/L, respectively. There were five parallels for each treatment. After two 50 g blank soil was spiked with 100 μL standard solution of 50 mg/L and 5 mg/L, respectively, it was evenly mixed and dried to reach the concentration of 0.1 mg/kg and 0.01 mg/kg. Another 50 g blank soil was spiked with 20 μL 5 mg/L standard solution to prepare the medicated soil of 0.002 mg/kg. The soil sample (50 g) for each treatment was divided into five equal parts for five parallels.

### Risk assessment

The risk quotient (RQ) model was used to evaluate the potential ecological risk of each pesticide to surface water and soil. If the non-observed effect concentration (NOEC) can be found in the database, it will be used preferentially for calculation. Otherwise, the EC_50_ or LC_50_ will be considered in this study according to the following calculation formula. Moreover, if no specific NOEC or EC_50_ value was given, the corresponding minimum value was used for RQ calculation.

$$ \begin{aligned} RQ & = \frac{MEC}{{PNEC}} \\ PNEC & = \frac{NOEC}{{100}}\quad {\text{or}}\quad PNEC = \frac{{E\left( L \right)C_{50} }}{1000} \\ \end{aligned} $$

where MEC was the actual monitoring concentration of pollutants, μg/L. PNEC was the predicted no effect concentration, μg/L. PNEC derived from the lowest toxicity values that observed for the most sensitive organisms (namely, the non-observed effect concentration, NOEC). EC_50_ was median lethal concentration. From the pesticide characteristic database (PPDB, https://sitem.herts.ac.uk/aeru/ppdb/), this study collected the acute toxicological data (EC_50_, assessment factor 1000) or chronic toxicological data (NOEC, assessment factor 100) of aquatic organisms at three nutrient levels, involving the primary producer (algae), primary consumer (aquatic invertebrates), and secondary consumers (fish) (Supplementary Table 13). Meanwhile, the acute (14 d-LC_50_) or chronic (NOEC) toxicological data of earthworm were collected and used to evaluate the pollution degree of these pesticides to soil. The potential aquatic and terrestrial ecological risks of eight pesticides were assessed by RQ model. The situation of 0.01 ≤ RQ < 0.1 means that the risk of pesticide to the target organism is lower, whilst the 0.1 ≤ RQ < 1 represents moderate risk. RQ ≥ 1 indicates the high risk of pesticide to the target organism.

Considering the coexistence of pesticides in environment, the cumulative risk quotient (*RQ*_all_) of multiple pesticides at each sampling position was also calculated according the following formula. Where, *RQ*_i_ was the risk quotient of each pesticide^22^.

$$ RQ_{all} = \mathop \sum \limits_{i = 1}^{n} RQ_{i} $$

### SOS/umu experiment

The autumn soil and water samples in autumn were pre-treated following the procedure section sample pre-treatment, the residuum was redissolved in sterile water to prepare the tested solution of SOS/umu experiment. (1) Strain culture: 20 μL bacterium solution (TA 1535/PSK 1002 strain) was inoculated in a sterile test tube holding 20 mL TGA culture medium and 20 μL ampicillin. The bacteria were cultured overnight at 37 °C, 180–190 rpm by a constant temperature incubator. The overnight culture broth was diluted 10 times with TGA medium using a sterile 100 mL-wide-mouth flask, and continuously incubated for 1.5 h under the same conditions. The bacterial broth in logarithmic growth phase was used in the following exposure experiment. (2) Exposure test. The 4-nitroquinoline-1-oxide (4-NQO), Dimethyl sulfoxide (DMSO), and sterile ultrapure water were used as positive control (PC), solvent control (SC), and negative control (NC), respectively. The ingredients for different treatments A-plate were as follows. SC treatment (H1–H6): 153 μL sterile ultrapure water + 27 μL 30% DMSO solution + 70 μL bacterial broth + 20 μL 10 × TGA medium. PC treatment (H7–H12): 153 μL sterile ultrapure water + 27 μL diluted 2000-times 4-NQO stock solution (1 mg/mL, 30% DMSO as solvent) + 70 μL bacterial broth + 20 μL 10 × TGA medium. NC treatment (A7–A12): 180 μL sterile ultrapure water + 70 μL bacterial broth + 20 μL 10 × TGA medium. Blank control (BK) treatment (A1–A6): 180 μL sterile ultrapure water + 70 μL TGA medium + 20 μL 10 × TGA medium. The plate-A covered with a lid was incubated at 37 ± 1 °C, 120–150 rpm for 2 h. The micropores of another plate B were filled with 270 μL TGA medium, and preincubated at 37 ± 1 °C. Subsequently, 30 μL culture solution for each micropore in plate-A was transferred to the corresponding micropores of plate-B, and incubated at 37 ± 1 °C, 120–150 rpm for 2 h. The absorbance value OD_600_ was measured by Thermo Scientific Microplate Reader. For another new plate-C, 120 μL B-buffer solution was added to each micropore, and preincubated at 28 °C. Afterwards, 30 μL culture solution for each micropore in plate-B was transferred to the corresponding micropores of plate-C, immediately added with 30 μL 2-nitrophenyl-β-D-galactopyranoside (ONPG) solution and mixed well. After the plate-C was incubated at 28 °C for another 30 min, immediate addition of 10% Na_2_CO_3_ iniferter (120 μL) was performed to terminate the reaction. The absorbance value OD_420_ was measured by Microplate Reader. The bacterial growth factor (G) and induction rate (IR) were calculated according to the following formulas.

$$ \begin{aligned} G & = \frac{{A_{600,S} - A_{600,B} }}{{A_{600,N} - A_{600,B} }} \\ IR & = \frac{{A_{420,S} - A_{420,B} }}{{A_{420,N} - A_{420,B} }} \times \frac{1}{G} \\ \end{aligned} $$

where A_600, B_ (A_420, B_), A_600, N_ (A_420, N_), A_600, S_ (A_420, S_) are the OD_600_ (OD_420_) values of blank control, negative control, and the tested sample, respectively. G ≤ 0.5 means that the samples exhibit cytotoxicity, in which it will be failed to calculate IR. The IR value was valid only if G is greater than 0.5. The 1.5 ≤ IR < 2 shows a suspected positive, whilst IR > 2 represents mutation positive. It is worth noting that the absorbance values (A_600,N_ and A_420,N_) of solvent control were used to calculate the G and IR of positive control.

To predict the potential carcinogenic risk of water samples from Fuhe river, the risk coefficient (P) was evaluated on basis of SOS/umu experimental result as the following formula.

$$ \begin{aligned} & TEQ_{4 - NQO} = \frac{{IR_{s} }}{{IR_{4 - NQO} }} \\ & P = \frac{{TEQ_{4 - NQO} \times 0.001 \times 2.0}}{65} \times 0.369 \\ \end{aligned} $$

TEQ_4-NQO_ (μg/L) was the equivalent concentration of the positive control, using to compare the toxicity of different water samples. 0.369 (unit, kg·day^−1^·mg^−1^) is the carcinogenic intensity of 4-NQO. The IR_S_ and IR_4-NQO_ are the induction rates of water sample and positive control, respectively. 0.001 is the unit conversion coefficient. The 65 kg and 2.0 L·day^−1^ are the adult average weight and daily water intake, respectively. The control standard of carcinogenic risk induced by chemicals should be in the range of 10^−6^–10^−4^. Considering the maximum risk of drinking water, 10^−6^ was used as the safe drinking standard reference in this study (USEPA-2008).

## Supplementary Information

Supplementary Tables.
