# Author Correction: Residue level, occurrence characteristics and ecological risk of pesticides in typical farmland-river interlaced area of Baiyang Lake upstream, China

**DOI:** 10.1038/s41598-022-17817-5

**Published:** 2022-08-09

**Authors:** Xiaoli Sun, Miao Liu, Jianwei Meng, Liping Wang, Xiaoxin Chen, Shan Peng, Xin Rong, Lei Wang

**Affiliations:** 1 Hebei Key Laboratory of Close‑to‑Nature Restoration Technology of Wetlands, School of Eco‑Environment, Hebei University, Baoding, 071002 Hebei Province People’s Republic of China; 2 College of Chemistry and Environmental Science, Hebei University, Baoding, 071002 Hebei Province People’s Republic of China; 3 Hebei Key Laboratory of Mineral Resources and Ecological Environment Monitoring, Hebei Research Center for Geoanalysis, Baoding, 071002 Hebei Province People’s Republic of China

Correction to: *Scientific Reports* 10.1038/s41598-022-16088-4, published online 14 July 2022

In the original version of this Article a previous rendition of Figure 2 was published. The original Figure 2 and accompanying legend appear below.

**Figure 2:**
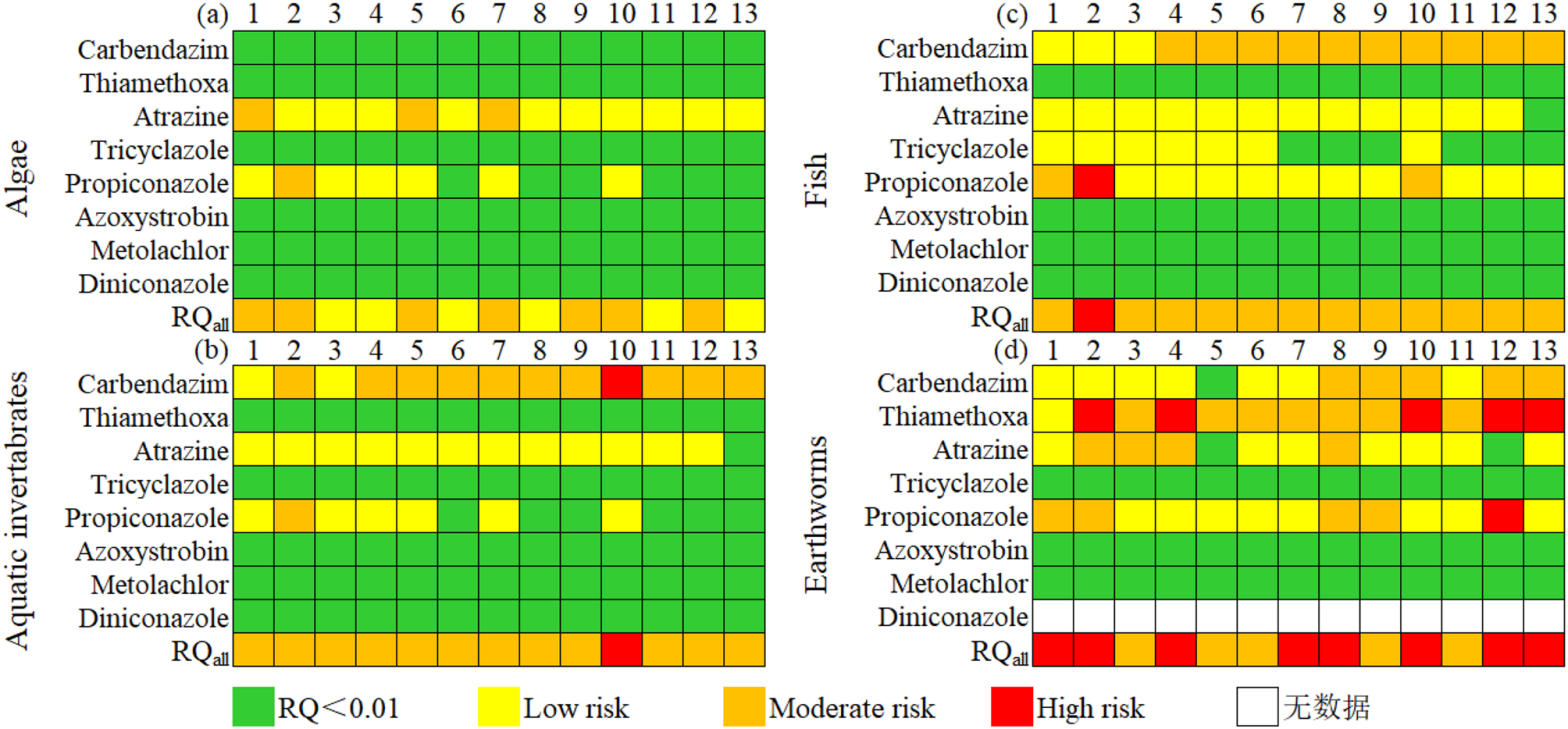
Heat map of RQ values of pesticides to algae (**a**), aquatic invertebrate (**b**), fish (**c**), and earthworm (**d**). Most of pesticides almost have no aquatic risk (RQ < 0.01), but carbendazim and propionazole deserved attention. The RQ_all_ were in the range of 0.4541–3.327 (earthworm), 0.0239–0.4552 (algae), 0.1094–1.103 (aquatic invertabrates), and 0.1657–1.923 (fish), respectively.

The original Article has been corrected.

