# Supplementary material for: Residue level, occurrence characteristics and ecological risk of pesticides in typical farmland-river interlaced area of Baiyang Lake upstream, China
Source: Sci Rep. 2022 Jul 14;12:12049. doi: 10.1038/s41598-022-16088-4 (PMC9283526; doi:10.1038/s41598-022-16088-4)
Supplement: Supplementary file 1 — Supplementary Tables. [file 41598_2022_16088_MOESM1_ESM.pdf]

Supplementary Table 1. RQ values of eight pesticides in autumn-waters to algae.

Waters	Carbendazim	Thiamethoxam	Atrazine	Tricyclazole	Propiconazole	azoxystrobin	Metolachlor	Diniconazole
SP1	≪0.01	≪0.01	0.1243	≪0.01	0.0395	≪0.01	≪0.01	0.0012
SP2	≪0.01	0.0015	0.0723	≪0.01	0.3779	≪0.01	≪0.01	0.0032
SP3	≪0.01	≪0.01	0.0574	≪0.01	0.0190	≪0.01	≪0.01	≪0.01
SP4	≪0.01	0.0056	0.0774	≪0.01	0.0121	≪0.01	≪0.01	≪0.01
SP5	≪0.01	0.0020	0.1068	≪0.01	0.0105	≪0.01	≪0.01	≪0.01
SP6	≪0.01	0.0027	0.0538	≪0.01	0.0079	≪0.01	≪0.01	≪0.01
SP7	≪0.01	0.0033	0.1380	≪0.01	0.0141	≪0.01	≪0.01	0.0037
SP8	≪0.01	0.0065	0.0466	≪0.01	0.0083	≪0.01	≪0.01	≪0.01
SP9	≪0.01	0.0048	0.0939	≪0.01	0.0090	≪0.01	≪0.01	≪0.01
SP10	≪0.01	0.0072	0.0503	≪0.01	0.0647	≪0.01	≪0.01	0.0020
SP11	≪0.01	0.0059	0.0488	≪0.01	0.0078	≪0.01	≪0.01	0.0061
SP12	≪0.01	0.0058	0.0937	≪0.01	0.0054	≪0.01	≪0.01	≪0.01
SP13	≪0.01	0.0059	0.0134	≪0.01	0.0039	≪0.01	≪0.01	≪0.01

Supplementary Table 2. RQ values of eight pesticides in autumn-waters to aquatic invertebrates.

Waters	Carbendazim	Thiamethoxam	Atrazine	Tricyclazole	Propiconazole	azoxystrobin	Metolachlor	Diniconazole
SP1	0.0578	≪0.01	0.0497	≪0.01	0.0407	0.0024	≪0.01	≪0.01
SP2	0.2071	≪0.01	0.0289	≪0.01	0.3901	0.0011	≪0.01	≪0.01
SP3	0.0644	≪0.01	0.0228	≪0.01	0.0197	0.0019	≪0.01	≪0.01
SP4	0.7917	≪0.01	0.0310	≪0.01	0.0125	0.0035	≪0.01	≪0.01
SP5	0.2761	≪0.01	0.0427	≪0.01	0.0108	0.0017	≪0.01	≪0.01
SP6	0.3873	≪0.01	0.0215	≪0.01	0.0081	0.0014	≪0.01	≪0.01
SP7	0.4601	≪0.01	0.0552	≪0.01	0.0146	0.0011	≪0.01	≪0.01
SP8	0.9194	≪0.01	0.0187	≪0.01	0.0085	0.0011	≪0.01	≪0.01
SP9	0.6720	≪0.01	0.0375	≪0.01	0.0093	0.0011	≪0.01	≪0.01
SP10	1.013	≪0.01	0.0201	≪0.01	0.0668	0.0022	≪0.01	≪0.01
SP11	0.8428	≪0.01	0.0195	≪0.01	0.0081	0.0015	≪0.01	≪0.01
SP12	0.8795	≪0.01	0.0375	≪0.01	0.0055	0.0011	≪0.01	≪0.01
SP13	0.8374	≪0.01	0.0054	≪0.01	0.0040	≪0.01	≪0.01	≪0.01

Supplementary Table 3. RQ values of eight pesticides in autumn-waters to fish.

Waters	Carbendazim	Thiamethoxam	Atrazine	Tricyclazole	Propiconazole	azoxystrobin	Metolachlor	Diniconazole
SP1	0.0271	≪0.01	0.0621	0.0204	0.1857	≪0.01	≪0.01	≪0.01
SP2	0.0971	≪0.01	0.0362	0.0103	1.779	≪0.01	≪0.01	≪0.01
SP3	0.0302	≪0.01	0.0287	0.0162	0.0896	≪0.01	≪0.01	≪0.01
SP4	0.3711	0.0028	0.0387	0.0283	0.0569	0.0010	≪0.01	≪0.01
SP5	0.1294	≪0.01	0.0534	0.0111	0.0492	≪0.01	≪0.01	≪0.01
SP6	0.1815	0.0014	0.0269	0.0111	0.0371	≪0.01	≪0.01	≪0.01
SP7	0.2157	0.0016	0.0690	0.0081	0.0663	≪0.01	≪0.01	≪0.01
SP8	0.4310	0.0033	0.0233	0.0088	0.0388	≪0.01	≪0.01	≪0.01

SP9	0.3150	0.0024	0.0469	0.0085	0.0424	≪0.01	≪0.01	≪0.01
SP10	0.4746	0.0036	0.0252	0.0146	0.3044	≪0.01	≪0.01	≪0.01
SP11	0.3950	0.0030	0.0244	0.0099	0.0368	≪0.01	≪0.01	≪0.01
SP12	0.4123	0.0029	0.0468	0.0090	0.0252	≪0.01	≪0.01	≪0.01
SP13	0.3925	0.0030	0.0067	0.0075	0.0184	≪0.01	≪0.01	≪0.01

Supplementary Table 4. Cumulative risk quotient (RQ_{all}) of eight pesticides to algae, aquatic invertebrates, and fish.

Waters	Total residue (ng/L)	RQ_{all}(Algae)	RQ_{all}(Aquaticinvertabrates)	RQ_{all}(Fish)
SP1	298.9	0.1658	0.1513	0.2966
SP2	1436	0.4552	0.6277	1.923
SP3	169.9	0.0781	0.1094	0.1657
SP4	694.0	0.0967	0.8396	0.4990
SP5	344.0	0.1202	0.3320	0.2449
SP6	362.4	0.0653	0.4189	0.2585
SP7	521.4	0.1594	0.5317	0.3614
SP8	740.1	0.0627	0.9485	0.5057
SP9	1723	0.1085	0.7207	0.4157
SP10	994.4	0.1247	1.103	0.8233
SP11	690.9	0.0691	0.8734	0.4703
SP12	708.8	0.1056	0.9244	0.4967
SP13	633.6	0.0239	0.8484	0.4284

Supplementary Table 5. RQ and RQ_{all} values of eight pesticides in autumn-soils to earthworm.

Soils	Carbendazim	Thiamethoxam	Atrazine	Tricyclazole	Propiconazole	Azoxystrobin	Metolachlor	Diniconazole	RQ_{all}
SP1	0.0130	0.0465	0.0933	≪0.01	0.9797	≪0.01	≪0.01	--	1.134
SP2	0.0266	1.070	0.1268	≪0.01	0.1090	≪0.01	≪0.01	--	1.333
SP3	0.0212	0.4019	0.1153	≪0.01	0.0740	≪0.01	≪0.01	--	0.6131
SP4	0.0375	1.223	0.2825	≪0.01	0.0956	≪0.01	≪0.01	--	1.640
SP5	0.0082	0.4256	0.0055	≪0.01	0.0145	≪0.01	≪0.01	--	0.4541
SP6	0.0604	0.8529	0.0333	≪0.01	0.0302	0.0013	≪0.01	--	0.9783
SP7	0.0640	0.9514	0.0683	≪0.01	0.0577	≪0.01	≪0.01	--	1.142
SP8	0.1113	0.6847	0.1211	≪0.01	0.5300	≪0.01	≪0.01	--	1.448
SP9	0.1773	0.3368	0.0988	≪0.01	0.1124	≪0.01	≪0.01	--	0.7259
SP10	0.1621	1.495	0.0748	≪0.01	0.0596	≪0.01	≪0.01	--	1.792
SP11	0.0918	0.5037	0.0122	≪0.01	0.0659	≪0.01	≪0.01	--	0.6741
SP12	0.3907	1.682	0.0083	≪0.01	1.245	≪0.01	≪0.01	--	3.327
SP13	0.1966	1.459	0.0229	≪0.01	0.0602	0.0010	≪0.01	--	1.740

Note: "--" means that there was no relevant toxicity data.

Supplementary Table 6. Growth factor, induction rate, and carcinogenic risk coefficient of surface water (20 times enrichment).

Waters	G	IR	$TEQ_{4\text{-}NQO}$ (μg/L)	P
SP1	1.06	1.32	0.498	5.66×10^{-6}
SP2	0.994	1.26	0.477	5.42×10^{-6}
SP3	0.988	1.35	0.512	5.82×10^{-6}
SP4	1.05	1.14	0.433	4.92×10^{-6}
SP5	1.05	1.12	0.425	4.82×10^{-6}
SP6	1.09	1.13	0.428	4.86×10^{-6}
SP7	1.06	1.14	0.432	4.90×10^{-6}
SP8	1.11	1.21	0.458	5.20×10^{-6}
SP9	1.05	1.23	0.465	5.28×10^{-6}
SP10	1.04	1.31	0.497	5.64×10^{-6}
SP11	1.04	1.37	0.518	5.88×10^{-6}
SP12	1.07	1.19	0.450	5.11×10^{-6}
SP13	1.03	1.21	0.459	5.21×10^{-6}
Positive control	0.794	2.64	--	--

Supplementary Table S7. Growth factor, induction rate, and potential carcinogenic risk coefficient (P) of soil.

Soils	G	IR	$TEQ_{4\text{-}NQO}$ (μg/L)
SP1	0.041	--	--
SP2	0.825	1.97	0.401
SP3	0.619	1.85	0.376
SP4	0.738	1.76	0.358
SP5	0.536	3.98	0.808
SP6	0.816	1.48	0.301
SP7	0.877	1.41	0.287
SP8	0.880	1.63	0.331
SP9	0.854	1.57	0.318
SP10	0.765	1.59	0.324
SP11	0.796	1.58	0.321
SP12	0.852	1.58	0.322
SP13	0.744	1.86	0.378
Positive control	0.607	4.92	--

Note: “--” means that cell growth was inhibited with no cytotoxicity.

Supplementary Table 8. Longitude and latitude of sampling positions.

Locations	Longitudes	Latitudes
SP1	115°59’14.27”	38°83’81.38”
SP2	115°58’17.80”	38°83’50.98”
SP3	115°60’20.99”	38°82’49.13”
SP4	115°61’73.17”	38°80’95.45”

SP5	115°62’44.63”	38°81’27.90”
SP6	115°65’36.91”	38°82’83.82”
SP7	115°74’52.81”	38°86’61.30”
SP8	115°74’30.32”	38°87’45.34”
SP9	115°78’78.38”	38°88’89.32”
SP10	115°82’10.37”	38°88’33.68”
SP11	115°86’93.76”	38°89’56.97”
SP12	115°91’02.46”	38°92’29.55”
SP13	115°93’54.77”	38°90’50.43”

Supplementary Table 9. Physical parameters of water and soil samples at 13 sampling positions.

Locations	Season	pH (Water)	DO (mg/L)	Temperature (°C)	pH (Soil)
SP1	Spring, Summer	8.29, 8.33	9.00, 7.29	16.8, 24.2	8.81, 9.05
	Autumn, Winter	7.21, 7.47	9.47, 11.41	16.4, 6.8	7.25, 8.25
SP2	Spring, Summer	8.36, 9.25	9.28, 9.12	13.8, 26.1	8.68, 8.62
	Autumn, Winter	8.27, 8.37	9.02, 14.60	14.9, 1.1	8.00, 8.43
SP3	Spring, Summer	8.43, 8.58	8.98, 8.62	16.0, 24.5	8.63, 8.36
	Autumn, Winter	7.42, 7.51	8.64, 10.75	16.6, 5.3	7.96, 7.81
SP4	Spring, Summer	8.99, 8.60	12.96, 8.08	14.2, 24.4	8.73, 9.07
	Autumn, Winter	8.86, 7.80	13.35, 4.16	12.1, 0.2	8.33, 8.62
SP5	Spring, Summer	8.17, 8.35	9.04, 8.10	16.5, 25.8	8.36, 8.97
	Autumn, Winter	7.88, 7.68	9.11, 10.66	16.4, 4.8	8.76, 8.46
SP6	Spring, Summer	8.42, 7.96	8.51, 7.83	17.9, 25.4	8.57, 8.42
	Autumn, Winter	8.13, 7.66	9.18, 10.61	15.4, 5.0	8.39, 8.29
SP7	Spring, Summer	8.34, 8.55	7.68, 8.58	17.8, 26.6	5.85, 8.70
	Autumn, Winter	8.01, 7.79	8.11, 10.72	15.8, 5.7	8.41, 8.35
SP8	Spring, Summer	8.30, 8.35	7.85, 6.78	17.10, 26.6	8.85, 8.63
	Autumn, Winter	7.95, 7.77	8.31, 11.15	15.7, 4.3	8.46, 8.42
SP9	Spring, Summer	8.43, 8.92	8.06, 13.50	19.0, 26.6	8.18, 8.56
	Autumn, Winter	8.15, 7.74	9.27, 10.45	16.3, 3.7	8.01, 7.99
SP10	Spring, Summer	8.68, 8.74	11.56, 11.60	19.1, 26.5	8.96, 8.89
	Autumn, Winter	8.19, 7.81	8.48, 12.72	16.4, 3.6	8.36, 8.67
SP11	Spring, Summer	8.58, 8.60	10.64, 8.31	17.7, 26.2	8.37, 8.68
	Autumn, Winter	8.18, 7.87	7.47, 10.88	16.2, 3.2	8.2, 8.52
SP12	Spring, Summer	8.51, 8.70	11.71, 7.31	18.4, 26.1	8.82, 8.48
	Autumn, Winter	8.08, 7.79	6.99, 13.13	15.2, 2.2	8.29, 8.33
SP13	Spring, Summer	7.94, 8.51	7.67, 6.77	19.9, 26.8	8.77, 8.86
	Autumn, Winter	8.06, 8.65	6.69, 5.45	18.5, 3.4	9.02, 8.43

Supplementary Table 10. Limits of detection and quantification of eight pesticides

Pesticides	Water		Soil	
	LOQ (μg/L)	LOD (μg/L)	LOQ (μg/L)	LOD (μg/L)
Carbendazim	1.06×10^{-2}	3.17×10^{-3}	4.64×10^{-2}	1.39×10^{-2}
Thiamethoxam	3.48×10^{-2}	1.04×10^{-2}	1.63	0.49
Atrazine	5.85×10^{-2}	9.24×10^{-3}	3.21×10^{-2}	9.62×10^{-3}
Tricyclazole	5.87×10^{-3}	4.45×10^{-3}	1.21×10^{-2}	3.63×10^{-3}
Propiconazole	5.83×10^{-3}	1.75×10^{-3}	1.36×10^{-2}	4.08×10^{-3}
Azoxystrobin	2.41×10^{-3}	9.28×10^{-4}	1.58×10^{-3}	4.73×10^{-4}
Metolachlor	7.10×10^{-3}	2.25×10^{-3}	7.97×10^{-3}	2.39×10^{-3}
Diniconazole	1.33×10^{-2}	2.93×10^{-3}	3.44×10^{-2}	1.03×10^{-2}

Supplementary Table 11. Instrument parameters of eight pesticides (LC-MS/MS).

Pesticides	Formulas	R.T. (min)	Precursor ion (m/z)	Quantification ion (m/z) Collision energy (V)	Confirmatory ion (m/z) Collision energy (V)
Carbendazim	$C_9H_9N_3O_2$	1.10	192.2	160.1/25	132/40
Thiamethoxam	$C_8H_{10}ClN_5O_3S$	1.04	292.1	181/30	211/30
Atrazine	$C_8H_{14}ClN_5$	2.88	216	174/22	96/22
Tricyclazole	$C_9H_7N_3S$	2.30	190.1	163.1/25	136.1/30
Propiconazole	$C_{15}H_{17}Cl_2N_3O_2$	3.67	342.1	159.1/35	69/20
Azoxystrobin	$C_{22}H_{17}N_3O_5$	3.22	404.3	372.2/15	344.2/20
Metolachlor	$C_{15}H_{22}ClNO_2$	3.64	284	252.2/20	176.3/20
Diniconazole	$C_{15}H_{17}Cl_2N_3O$	3.63	326.1	70/30	158.7/30

Supplementary Table 12. Elution method of eight pesticides chromatographic conditions (LC-MS/MS).

Pesticides	R.T. (min)	Mobile phase A (%)
Carbendazim, Thiamethoxam	0.50	95
	1.50	Stop
Atrazine, Tricyclazole, Propiconazole, Azoxystrobin, Metolachlor, Diniconazole	0.50	25
	1.00	60
	3.50	95
	4.00	95
	4.10	25
	5.00	Stop

Supplementary Table 13. Acute or chronic toxicological data of eight pesticides to aquatic organisms and earthworm.

Pesticides	Algae (μg/L)	Aquatic invertebrates (μg/L)	Fish (μg/L)	Earthworm (mg/kg)
Carbendazim	NOEC 7.7×10^3	NOEC 1.5	NOEC 3.2	NOEC 1.0
Thiamethoxam	EC_{50} $>1.0\times10^5$	NOEC $>1.0\times10^5$	NOEC 2×10^4	NOEC 5.34
Atrazine	NOEC 100	NOEC 250	NOEC 2×10^3	LC_{50} 79
Tricyclazole	EC_{50} 8.2×10^3	NOEC 960	NOEC 81	NOEC 250
Propiconazole	NOEC 320	NOEC 310	NOEC 68	NOEC 0.833
Azoxystrobin	NOEC 800	NOEC 44	NOEC 147	NOEC 3.0
Metolachlor	NOEC 3.0×10^3	NOEC >707	NOEC 1.0×10^3	LC_{50} 140
Diniconazole	NOEC 72	NOEC 3.59×10^3	NOEC 1.71×10^3	--

Note: "--" means that there was no relevant toxicity data. If no definite NOEC or EC_{50} value was given, the corresponding minimum value was used for RQ calculation.
